# Hyperuricemic Renal Failure in Nonhematologic Solid Tumors: A Case Report and Review of the Literature

**DOI:** 10.1155/2012/314056

**Published:** 2012-05-27

**Authors:** Neeraj Saini, Kyeong Pyo Lee, Smita Jha, Sanket Patel, Neelima Bonthu, Ankit Kansagra, Ashmeet Bhatia, Sandra E. Martinez, Jaymin Patel, Sarah Altamimi, Sara Ghotb

**Affiliations:** Department of Internal Medicine, North Shore Medical Centre (NSMC), Salem, MA 01970, USA

## Abstract

Tumor lysis syndrome (TLS) is an oncologic emergency that is caused by massive tumor cell lysis. It is commonly associated with hematological cancers like leukemia and lymphoma and uncommonly with solid nonhematologic tumors as well. However, spontaneous tumor lysis syndrome (STLS) without any cytotoxic chemotherapy rarely occurs in solid tumors. We describe a case of STLS in a metastatic adenocarcinoma of unknown primary and review the literature of STLS in solid non-hematologic tumors to identify various risk factors for pathogenesis of this entity.

## 1. Case Report

**Figure 1:**
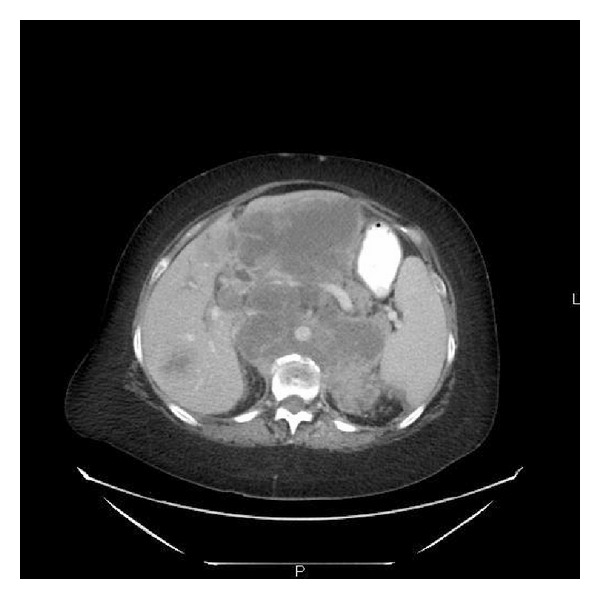
Computed tomography of abdomen showing a big retroperitoneal mass engulfing the vascular structures in the abdomen.

A 59-year-old Caucasian female with past medical history of hypertension, obesity, mild osteopenia, and >20 pack years smoking history presented to the primary care physician with a more than four-month history of generalized weakness, anorexia, weight loss of more than 30 pounds, a growing subcutaneous mass in the right lower back and back pain in the lumbar region. MRI of the spine revealed confluent bulky soft tissue mass measuring approximately 14 cm anteroposterior × 13 cm transverse in size in the retroperitoneum region and pathological compression fracture of L1 vertebrae. Further, CT scan of chest/abdomen/pelvis confirmed bulky retroperitoneal mass/adenopathy with extensive liver metastases and multiple tiny pulmonary nodules (Figure 1).

The mass at lower back was excised and immunohistochemical staining of subcutaneous mass was positive for cytokeratin 7, cytokeratin 20, mucicarmine and villin but negative for ER, mammaglobin, TTF-1, napsin A, CDX2, p63, calretinin, and hepatocyte antigen. The villin positivity in conjunction with the cytokeratin 7 positive expression is suggestive of a noncolorectal gastrointestinal origin, including pancreaticobiliary/gallbladder source. The tissue of origin assay was unable to locate the primary source and subsequently, diagnosis of poorly differentiated adenocarcinoma of unknown primary was rendered. Ten days after the biopsy, while waiting for chemotherapy to begin, patient presented to her primary care physician with severe nausea and vomiting, altered mental status and decreased urine output. Basic metabolic panel (Chem-7) was consistent with dehydration with blood urea nitrogen (BUN) and creatinine of 31 mg/dL and 1.6 mg/dL, respectively, elevated from her baseline normal values about 10 days later. Patient was given a liter of intravenous fluid and was sent back home with antiemetics.

Four days later, after nausea and vomiting persisted with lethargy, weakness and very poor appetite, patient presented to emergency room. Chem-7 revealed BUN of 117 mg/dL with a creatinine of 7.5 mg/dL. Other pertinent lab values with normal values in parentheses showed calcium 6.5 (8.9–10.3) mg/dL, phosphorus 8.8 (2.6–4.6) mg/dL, uric acid 26.5 (2.8–6.6) mg/dL, and lactate dehydrogenase 1265 IU/L with normal transaminases. Obstructive uropathy was excluded by retroperitoneal ultrasound. Patient went into oliguric acute renal failure (ARF) and was treated with intravenous fluids. However, renal function did not improve over the next two days, and hemodialysis was started along with rasburicase. Lab abnormalities were consistent with TLS. Due to poor prognosis, patient was not started on chemotherapy and discharged to hospice without chemotherapy, and she died three weeks later.

## 2. Discussion

Tumor lysis syndrome (TLS) is an oncologic emergency that is caused by massive tumor cell lysis with the release of large amounts of potassium, phosphate, and nucleic acids into the systemic circulation. The Cairo-Bishop definition of TLS [1], proposed in 2004, provided both clinical and laboratory diagnostic and grading criteria for TLS as per below:

- laboratory TLS is defined as either a 25% change or level above or below normal for any two or more serum values of uric acid, potassium, phosphate, and calcium within 3 days before or 7 days after the initiation of chemotherapy in the setting of adequate hydration and without use of any uricosuric agent;
- clinical TLS is defined as laboratory TLS plus one or more of the following that was not directly attributed to a therapeutic agent: increased serum creatinine (≥1.5 times the upper limit of the normal, cardiac arrhythmia/sudden death, or a seizure);

TLS most often occurs after the initiation of cytotoxic therapy in patients with high-grade lymphomas (particularly the Burkitt subtype) and acute lymphoblastic leukemia. TLS has been rarely described after treatment of nonhematologic solid tumors including breast cancer, germ cell tumors [2], small-cell carcinoma (mostly involving the lung) [2, 3], medulloblastoma [2], sarcoma [2], metastatic colorectal cancer, gastrointestinal stromal tumors, melanoma [2], hepatocellular carcinoma [2], and other solid tumors as well. TLS has been shown to occur spontaneously without any cytotoxic therapy in hematologic cancers; however, it is very rare to find spontaneous tumor lysis syndrome (STLS) in solid tumors. The extensive search of Pubmed for STLS in non-hematologic solid tumors yielded 15 case reports including our case report (Table 1).

We believe that our case had STLS because of evidence of necrosis in the tumor and was probably precipitated by the dehydration aggravated due to nausea and vomiting. We excluded other causes for ARF, such as postrenal obstruction (by ultrasound and CT scan), urinary tract infection (urinalysis was normal for WBCs), parenchymal kidney disease (which does not develop so quickly and renal parenchyma appeared morphologically normal on ultrasound and CT scan). Elevated serum uric acid is present in acute renal failure of any cause, especially in prerenal azotemia, but values are generally below <15 mg/dL except in TLS. In our patient, serum uric acid >25 mg/dL was again consistent with TLS. The typical laboratory findings in outpatient such as hyperuricemia, hyperkalemia, hypocalcemia, hyperphosphatemia, and ARF met the laboratory and clinical criteria of TLS.

According to TLS expert panel guidelines [15], most solid tumors are classified as low risk for TLS, whereas tumors which are sensitive to chemotherapy or have high-proliferative index, such as neuroblastomas, germ cell, and small-cell lung cancers, are classified as intermediate risk. One-third of cases in Table 1 belonged to this intermediate risk group, notably germ cell tumors [4, 5, 10], coherent with the fact that highly proliferative tumors have more tendency to form large masses and presents with significant tumor burden offering them a more likelihood to induce STLS.

The risk factors for STLS in solid tumors, based on the review of literature and these case reports, include chemotherapy sensitive or high proliferative index tumors, tumor burden, presence of liver and bone metastasis, abnormal lab values including elevated serum uric acid, serum LDH, and concomitant presence of risk factors for renal failure. The most important risk factor was the tumor burden with every report documenting stage 4 cancers having extensive liver and bony metastases and presence of big-sized masses (>10 cm), which bestow them high predilection to undergo autonecrosis releasing intracellular contents. The biopsy in most of the cases revealed necrotic tissue in the center of the masses as well as in liver and bone metastases. The metastases in liver and bone compared to an abdominal mass are at higher risk to undergo ischemic central auto-necrosis due to pressure induced by surrounding normal parenchyma on a fast growing tumor, whereas, an intra-abdominal mass like in retroperitoneal region can grow to a significant size without lysis.

Another important risk factor would be preexistent impaired renal function that can be further compromised with the progression of the syndrome. The renal function deterioration can lead to deficient clearance of elevated uric acid produced by tumor lysis and deposition of the crystals in renal tubules (uric acid nephropathy). The use of nephrotoxic agents like intravenous contrasts agents, nonsteroidal anti-inflammatory drugs, nephrotoxic antibiotics and drugs that inhibit uric acid secretion (thiazides, and aspirin) should be avoided.

STLS is not as common in solid tumors compared to hematologic malignancies, but we believe that it might be an underdiagnosed entity, as many cases of mild grade TLS with less deranged abnormalities in the laboratory parameters can be missed easily. The patients with above risk factors should be treated prophylactically with aggressive hydration and uricolytic agents before the initiation of chemotherapy. Our case highlights the criticality of identifying STLS in solid tumors with big tumor burden and treating prophylactically to prevent this cataclysmic syndrome.

## Figures and Tables

**Table 1:** Published Case reports of spontaneous tumor lysis syndrome in solid non-hematologic tumors published in the literature.

Age sex	Type of cancer	Primary site	Tumour burden and metastases	Lab values*		Treatment	Outcome
13, F [4]	Germ cell tumor–pure germinoma	Pineal and suprasellar mass	20 × 17 × 13 cm pelvic mass with numerous peritoneal deposits	LDH	2310	Rasburicase with hydration Platinum-based chemotherapy given.	Survived
				Uric acid	28		
				K; Ca; Ph	5.6; 7.2; 7.3		
				Creat	3.3		

22, M [5]	Germ cell tumour–choriocarcinoma	Testis	Retroperitoneal mass of 14 cm in diameter with massive Liver and Lung metastases and numerous lymph nodes	LDH	4055	Hydration and rasburicase	Died
				Uric acid	18		
				K; Ca; Ph	7.2; 9.6; 7.2		
				Creat	4.5		

53, M [6]	Squamous cell Carcinoma	Maxillary Sinus	2.8 × 2.2 × 2 cm papilloma in maxillary sinus with extensive metastases in liver	LDH	1000	Hydration; urinary alkalization with allopurinol and rasburicase	Died
				Uric acid	20.9		
				K; Ca; Ph	7.6; 6.2; 11.8		
				Creat	6.4		

74, M [7]	Squamous cell carcinoma	Lung	Stage 4	LDH	N/a	Hydration; uricolytic therapy with hemodialysis	Survived
				Uric acid	15.4		
				K; Ca; Ph	5.2; n/a; 4.7		
				Creat	4.7		

72, M [8]	Prostate carcinoma	Prostate	Extensive liver metastases and bone metastases	LDH	1288	Hydration; allopurinol, and daily hemodialysis	Died
				Uric acid	28.1		
				K; Ca; Ph	4.9; 8.0; 8.3		
				Creat	6.1		

82, F [9]	Colon carcinoma	Colon	Multiple large nonhomogenous liver masses with necrosis	LDH	2304	Hydration, alkalization, and allopurinol	Survived
				Uric acid	20.4		
				K; Ca; Ph	n/a; 5.7; 5,5		
				Creat	3.5		

80, M [9]	Pheochromocytoma	Adrenal glands	20 cm diameter mass on adrenal glands with central necrosis	LDH	964	Hydration, alkalization, and allopurinol	Survived
				Uric acid	16.5		
				K; Ca; Ph	6.6; 8.4; 5.8		
				Creat	2.8		

72, M [9]	Hepatocellular carcinoma	Liver	n/a	LDH	1024	Hydration, alkalization, and allopurinol	Died
				Uric acid	20.1		
				K; Ca; Ph	4.5; 7.2; 5.4		
				Creat	3.2		

52, M [10]	Germ cell tumor–endodermal Sinus tumor	n/a	Bulky para-aortic lymphadenopathy with liver and lung mets with necrotic tissue	LDH	13400	Hemodialysis and chemotherapy initiated	Survived but died 4 months later
				Uric acid	21.8		
				K; Ca; Ph	7.9; 5.0; 7.1		
				Creat	4.19		

24, M [10]	Germ cell tumor–seminoma	Testis	20 × 25 cm retroperitoneal mass with liver metastases	LDH	13070	Hemodialysis with chemotherapy initiated. Later surgical removal of residual mass	Survived
				Uric acid	24		
				K; Ca; Ph	8.5; 7.6; 10		
				Creat	5.06		

50, M [11]	Metastatic adenocarcinoma of unknown primary	Unknown primary	Extensive tumor nodules in the liver with liver extending 17 cms below the costal margin with bulky lymphadenopathy and vertebral metastasis	LDH	n/a	Hydration with alkalization and allopurinol	Died
				Uric acid	37		
				K; Ca; Ph	6.5; 8.3; 9.2		
				Creat	4.7		

62, F [12]	Inflammatory breast cancer–lobular carcinoma	Breast	Large breast mass and supraclavicular lymphadenopathy with multiple mets in bones, lung, liver, and bone marrow	LDH	509	Allopurinol and chemotherapy initiated	Survived but died of recurrence 16 months later
				Uric acid	10.1		
				K; Ca; Ph	n/a; 10.1; 6.0		
				Creat	0.9		

36, M [13]	Gastric cancer adenocarcinoma	Stomach	Huge mass of more than 7 cm with multiple hepatic mets and lymphadenopathies	LDH	13924	Hydration, alkalization, allopurinol, hemodialysis, and chemotherapy initiated	Died
				Uric acid	16.9		
				K; Ca; Ph	5.6; 7.0; 6.9		
				Creat	2.9		

72, M [14]	Lung cancer adenocarcinoma	Lung	Large left upper lobe mass with multiple liver mets	LDH	1016	Hydration, potassium and phosphate binders, calcium gluconate, and allopurinol	Died
				Uric acid	12.65		
				K; Ca; Ph	7.0; 8.2; 8.3		
				Creat	1.28		

*Lab values are given as mg/dL for uric acid, serum creatinine, Potassium, Calcium and Phosphate and as IU/mL for LDH.
